# Effect of Short Carbon Fiber Volume Fraction on High-Temperature Tensile Properties of SCF/2A12 Composite

**DOI:** 10.3390/ma18174143

**Published:** 2025-09-04

**Authors:** Jinhao Wu, Shiyin Huang, Qingnan Meng, Mu Yuan, Sifan Wang, Xinyue Mao, Yuting Qiu, Linkai He

**Affiliations:** 1 School of Chemical Engineering and Technology, China University of Mining and Technology, Xuzhou 221116, China; wu_jinhao@jxnhu.edu.cn; 2 Jiaxing Key Laboratory of Intelligent Manufacturing and Operation & Maintenance of Automotive Parts, College of Mechanical and Electrical Engineering, Jiaxing Nanhu University, Jiaxing 314001, China; 3 Key Laboratory of Drilling and Exploitation Technology in Complex Conditions, Ministry of Natural Resources, College of Construction Engineering, Jilin University, Changchun 130026, China; huangsy22@mails.jlu.edu.cn (S.H.); yuanmu21@mails.jlu.edu.cn (M.Y.); wangsf22@mails.jlu.edu.cn (S.W.); maoxy21@mails.jlu.edu.cn (X.M.); qiuyt23@mails.jlu.edu.cn (Y.Q.); 4 State Key Laboratory of Superhard Materials, Jilin University, Changchun 130026, China; 5 Key Laboratory of Rock Mechanics and Geohazards of Zhejiang Province, School of Civil Engineering, Shaoxing University, Shaoxing 312000, China

**Keywords:** metal matrix composites, short carbon fiber, tensile strength, load transfer

## Abstract

To meet the increasing performance requirements of drilling pipes, including a reduced weight and enhanced mechanical and thermal properties, the application of aluminum alloys must be further advanced. Short-carbon-fiber-reinforced 2A12 aluminum alloy composites were fabricated via powder metallurgy. The density, hardness, and tensile strength of the composites were measured. The influence of the carbon fiber content on the composite’s mechanical properties was investigated across various temperatures. The composite material exhibited maximum yield strengths of 412 MPa at room temperature, 381 MPa at 180 °C, and 337 MPa at 220 °C. Incorporating carbon fibers increased the service temperature of a 2A12 aluminum alloy by approximately 40 °C. The strength increment of composites with a fiber content below 6 vol.% corresponded to the load transfer mechanism of carbon fiber, while the reason for non-conformity at a more than 6 vol.% fiber content was the continuous fracturing of carbon fibers, leading to the failure of the composites.

## 1. Introduction

**Figure 1:**
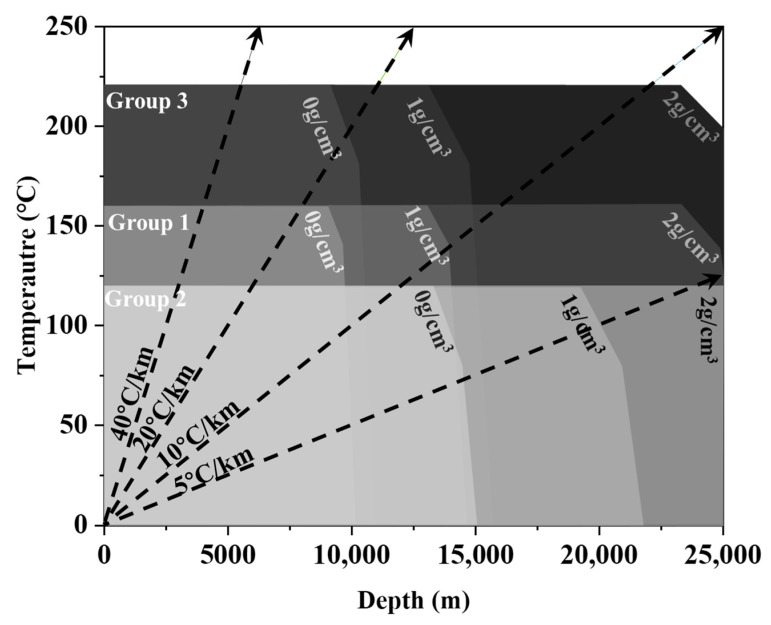
The operation temperature and depth of use of a typical aluminum alloy used in drill pipes. Group 1: Al-Cu-Mg; Group 2: Al-Zn-Mg; Group 3: Al-Cu-Mg-Si-Fe. The arrows represent the relationship between the depth and formation temperature under different geothermal gradients. The drill pipe is Φ147 mm × 11 mm with protector thickening and 11.8 m long, with a density of 3.271 g/cm^3^.

In drilling engineering, the weight per foot of drill string is a key design parameter, because the drill pipes bear the weight of the drill string [1]. Therefore, the use of a light alloy to replace the traditional steel drill pipe material is increasingly drawing attention [2]. A heat-treatable aluminum alloy has the characteristics of low density, good performance, and stable supply and has become an ideal material for drilling pipes in drilling engineering [3,4]. Hence, it has been widely used in scientific deep wells and oil and gas drilling wells [5,6]. The maximum operating temperature of the existing aluminum alloy grades for use in drill rods and their depth of use in drilling fluids of different densities are shown in Figure 1. The approximate relationship between the depth of the well and the formation temperature at different ground temperature gradients is also shown. The existing room-temperature high-strength aluminum alloy grades (Group 2, such as AA7075 and 1953T1) have the problem of poor high-temperature performance, while the existing higher-temperature-resistant aluminum alloy grades (Group 1, such as 2A12 and D16T) have the problem of low absolute strength [6,7].

In recent years, with the requirements posed by working conditions such as those involved in Moho drilling, geothermal well drilling, and space drilling, there are higher requirements for the service temperature and strength density ratio of drill pipes [5,8]. From Figure 1, it can be seen that the depth of use drops by thousands of meters when the temperature increases to the critical value.

Due to its high strength, low density, and favorable processability, carbon fiber (CF) is extensively employed as a reinforcing phase in metal matrix composites to enhance their mechanical behavior. Drill pipes are usually extruded with a variable cross-section, so short carbon fiber (SCF) is more suitable than continuous carbon fiber. Because of the non-wetting properties of carbon and aluminum, researchers have tried a variety of preparation methods. Akbarzadeh et al. [9] utilized the thixotropic nature of semi-solid slurry to fabricate a short-carbon-fiber-reinforced Al-Si alloy composite, reporting a 24% improvement in the tensile strength due to the incorporation of SCF. Li et al. [10] chose to use the mechanical stirring and sintering method to produce composites, resulting in a 53.6% increase in the tensile strength to 172 MPa. This improvement was attributed to the achievement of multi-orientation dispersion due to the crucible design. Choi et al. [11] prepared aluminum matrix composites via low-pressure infiltration using copper-coated short carbon fiber; the volume fraction of reinforcement increased to 30%. Jia et al. [12] used spark plasma sintering to fabricate a short-carbon-fiber-reinforced aluminum 6061–boron carbide hybrid composite, finding that SCF bore the main load and had a synergistic strengthening effect, thereby improving tensile strength and toughness. Zhou et al. [13] further investigated the effect of carbon fiber orientation on the mechanical properties and found that the composite had better performance when the composite was parallel to the carbon fiber plane. Jang et al. [14] prepared A356 composites with uncoated and SiC-coated short carbon fibers using a composite precursor followed by melt ultrasonication, and a significant increase in strength was only observed in the composite reinforced by the SiC-coated short carbon fiber. Szymanski [15] manufactured aluminum matrix composites through precision casting in a vacuum chamber and obtained a good-quality composite by adequately filling capillary spaces. Lv [16] fabricated a hybrid aluminum matrix through powder metallurgy, and the result showed that the tensile strength peaked at 207 MPa with 5% SiC particles and 7% carbon fiber.

Powder metallurgy is an effective method for improving high-temperature mechanical properties of aluminum alloys. For un-extruded samples, short carbon fiber could increase the yield strength (YS) and ultimate tensile strength (UTS) by 45.6% and 21.8% for aluminum alloy 2024 at 150 °C [17]. The improvement in the high-temperature performance of aluminum alloys by SCF was further verified through thermal exposure at 160 °C for 500 h [18]. Compared with AA2024, the composite still exhibited significant improvements in mechanical performance, with YS and UTS increasing by 100 MPa and 30 MPa, respectively. For samples subjected to hot pressing followed by hot extrusion, the reinforcing effect in 7075 aluminum alloy is not as good as that in 2024 aluminum alloy [19]. Enhancement in the ultimate tensile strength of the AA became evident only when the SCF volume fraction reached 12%, leading to respective increases of 15.6% at 150 °C and 16.5% at 200 °C. Even with TiO_2_ coating, short carbon fiber had less improvement in AA7075 than in AA2024 [20]. So, it remains unknown whether the hot extrusion reduces the strengthening effect or replacing different aluminum alloy substrates reduces the strengthening effect. However, how varying the volume fraction of short carbon fibers influences the high-temperature tensile behavior of the Al-Cu-Mg alloy after hot extrusion has not been fully explored.

This work aims to improve the high-temperature strength of 2A12 aluminum alloy by adding short carbon fibers. The effects of SCF content on tensile properties at different temperatures were studied, and the deviations between theoretical values and test values were discussed.

## 2. Materials and Methods

Aluminum alloy 2A12 (GB/T 3190) was selected as the matrix material. The powder, supplied by Jiweixin Metal Powder Co., Ltd. (Ningxiang, China), featured particle sizes ranging from 75 to 150 µm, and its chemical composition is detailed in Table 1. Desized Polyacrylonitrile-based carbon fibers (T700, Toray Co., Ltd., Tokyo, Japan) were subjected to ball milling to produce short carbon fibers. These fibers exhibited an aspect ratio below 42.8.

**Figure 2:**
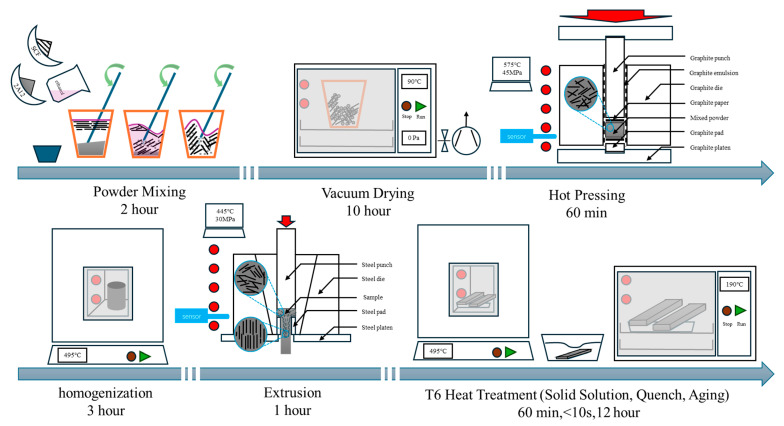
Flowcharts of the experimental procedures, equipment, and corresponding timelines. The condition of the aluminum alloy particles of carbon fiber is illustrated in the enlarged image.

Aluminum alloy powder was blended with sufficient anhydrous ethanol, and then short carbon fibers were added to the mixture. The mixture was manually stirred (for about two hours) to form a thixotropic fluid and then vacuum-dried at 90 °C for 10 h. Cylindrical specimens with a diameter of 30 mm were fabricated by applying hot pressing at 575 °C and 45 MPa pressure in a vacuum furnace for 60 min [21]. After homogenization at 495 °C for 3 h, the samples were extruded into flat plates at 445 MPa, and the extrusion ratio was 14.7 [22]. All the specimens were subjected to T6 heat treatment. The specific parameters were as follows: 495 °C × 1 h solid solution + water quenching + 190 °C × 1 h artificial aging [23]. A schematic diagram of experimental procedures is shown in Figure 2. The samples were sanded with sandpapers of different mesh sizes to remove burrs before testing. Composites with 2, 4, 6, 8, and 10 vol.% short carbon fibers were abbreviated as 2cf, 4cf, 6cf, 8cf, and 10cf, while the comparison samples 2A12 (named as 2A12) were prepared using the same procedure.

Sample densities were determined via Archimedes’ principle, whereas the theoretical density was computed based on the densities of carbon fiber (1.80 g/cm^3^) and 2A12 alloy (2.77 g/cm^3^). The microhardness tester (HXD-1000TM, Shanghai, China) was used to measure Vickers hardness under a 50 g load for 15 s, and the mean value from at least nine measurements was calculated. In line with the ASTM standard E−8/E8M−09, tensile samples with a gauge length of 10 mm were prepared. The dog-bone-shaped samples were processed from the flat plates by electrical discharge machining and then split into thin slices of about 2 mm. A universal testing machine (Sino Test Equipment Co., Ltd., Changchun, China) with a high-temperature atmosphere chamber was used to carry out tensile tests at room temperature, 180 °C, and 220 °C under a strain rate of 0.05 mm/min. The fiber length and distribution were observed using an optical microscope, and more than 300 short carbon fibers were measured by circular statistics. X-ray diffraction (XRD, DX2700, Dandong, China) using Cu Kα radiation was employed to identify the high-temperature phase constituents of the samples, scanning from 30° to 85° in step mode. The fracture morphology was recorded using a scanning electron microscope (SEM, S4800, Tokyo, Japan).

## 3. Results and Discussion

### 3.1. Mechanical Properties Enhanced by Addition of Carbon Fiber

The measured density ($\rho_{measured}$) of the 2A12 was 2.756 g/cm^3^, and the addition of short carbon fiber lowered the samples’ density, as shown in Table 2. When the volume fraction of SCF increased, the density of composite steadily dropped from 2.742 g/cm^3^ to 2.665 g/cm^3^. The relative density ($\rho_{relative}$) of the composite is calculated by

(1) $$\rho_{relative}=\frac{\rho_{measured}}{\rho_{f}V_{f}+\rho_{m}V_{m}}$$

where $\rho_{f}$ and $\rho_{m}$ are the theoretical density of the SCF and 2A12 matrix, respectively. $V_{f}$ and $V_{m}$ are the volume fractions of the SCF and matrix, respectively. All the samples’ relative density values exceed 99%, indicating that dense samples can be obtained through powder metallurgy.

**Figure 3:**
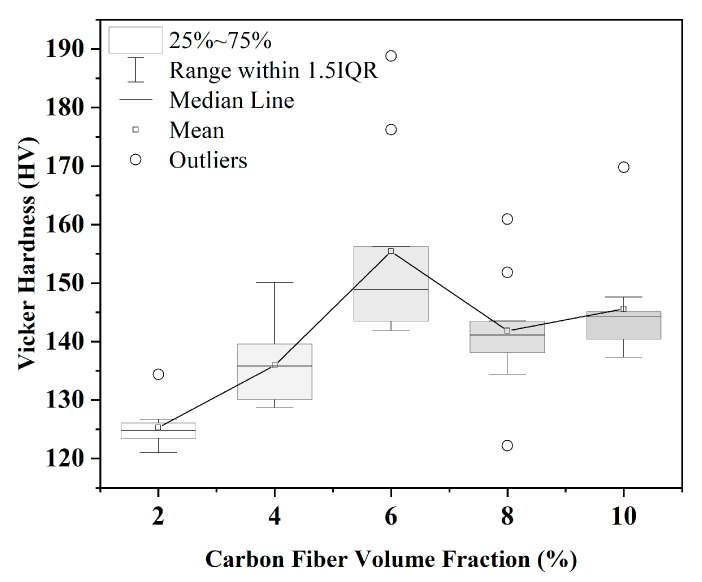
The Vickers hardness of composites with different carbon fiber volume fractions.

Figure 3 presents the Vickers hardness of composites with different short carbon fiber volume fractions. The mean hardness of the composites increased (with SD) from 125 HV to 155 HV as the volume fraction rose to 6%, as shown in Table 2. The improvement in hardness is mostly caused by the high modulus of carbon fiber at the surface and near the surface. The interquartile range is notably wider at 6%, indicating greater variability. Several outliers are present, particularly at 6% and 8%, and are considered to result from indentations coinciding with multiple short carbon fibers, leading to locally elevated hardness readings. Beyond this point, at 8% and 10%, the median hardness and mean hardness plateau. This trend was also reported in composites with pure Al [24] and pure Mg [25] matrices. This indicates that the reinforcement significantly enhances the hardness of the aluminum matrix up to a 6% volume fraction.

**Figure 4:**
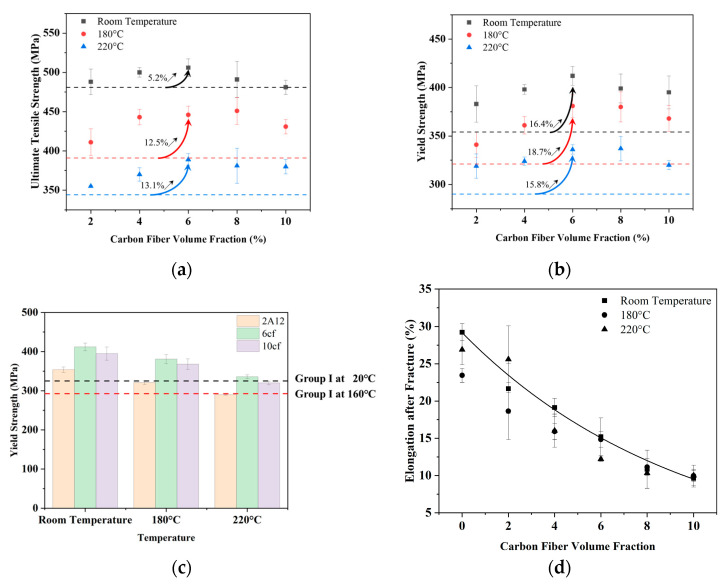
Tensile properties of SCF/2A12 and 2A12 samples. (**a**) Ultimate tensile strength; (**b**) yield strength; (**c**) the comparison of base metal (0%), optimum (6%), and over-doped (10%). (**d**) The elongation after fracture of SCF/2A12 and 2A12 samples. The dash line in (**a**,**b**) refers to the strength of 2A12 samples, and the black, red, and blue colors represent each temperature. The black dash line in (**c**) is the minimum yield strength of Group I material [6] at 20 °C. The red dash line in (**c**) is the minimum yield strength of Group I material at 160 °C. The black line in (**d**) is the trend line of composites at room temperature.

The ultimate tensile strength, yield strength, and fracture elongation of 2A12 and composites containing 2cf to 10cf at room temperature, 180 °C, and 220 °C are summarized in Figure 4. The UTS and YS of the matrix are demonstrated in Figure 4a,b by black, red, and blue dash lines for comparison. At room temperature, the ultimate tensile strength (UTS) of the composites initially increases from 488 MPa (2cf) to 506 MPa (6cf), representing a 5% improvement compared to the unreinforced matrix (481 MPa). However, the UTS drops back to the level of the matrix, just like the decreasing trend shown in Vickers hardness. At 180 °C, all samples show a noticeable reduction in UTS, while the matrix exhibits a lower strength of 391 MPa. The best performance is observed within 4~8% volume fraction, with UTS values fluctuating between 440 MPa and 450 MPa. Upon further increasing the testing temperatures to 220 °C, 6cf maintains the highest ultimate tensile strength (389 MPa), which is approximately 13% higher than the matrix (344 MPa). A cross-temperature comparison shows that the composite containing 6% carbon fiber at 220 °C exhibits a tensile strength higher than that of the unreinforced aluminum matrix at 180 °C, indicating that incorporating short carbon fibers effectively raises the service temperature of 2A12 aluminum alloy by approximately 40 °C.

The yield strength is similarly enhanced by the addition of short carbon fibers. At room temperature, it increases by nearly 30 MPa with 2 vol.% SCF addition, and then reaches 412 MPa at 6cf, which is 16% higher than the corresponding value of 2A12 (354 MPa), before slightly decreasing to 395 MPa at 10cf. This trend is largely preserved at elevated temperatures, with peak yield strengths of 381 MPa and 337 MPa for the 6cf and 8cf composites at 180 °C and 220 °C, respectively. At 180 °C, all composites except 2cf exhibit higher yield strength than the matrix at room temperature, as shown in Figure 4b. At 220 °C, only the yield strengths of the 6cf and 8cf samples were still higher than that of the matrix at 180 °C. However, the yield strength of other samples is equivalent to the matrix performance at 180 °C. These results indicate that the appropriate addition of carbon fiber not only suppresses the degradation of UTS and yield strength at elevated temperatures but also effectively extends the operational temperature window of the Al-Cu-Mg aluminum alloy by approximately 40 °C. From Figure 4c, it can be seen that the yield strength of optimal composites (6cf), over-doped composites (10cf), and base metal (2A12) samples are higher than the minimum yield strength of the Group I material at room temperature, and their elevated temperature properties are close to the critical strength at 160 °C. This means that the SCF/2A12 has the potential to cover the application scope of group 3 material (Figure 1).

Short carbon fiber reinforcement results in reduced elongation of the composites. The elongation of 2A12 is about 26.5%, while the elongations of 8cf and 10cf are about 10.0%. Variation in test temperature only slightly impacts the elongation of the composite, for the results of high temperatures are identical to the trend line of composites at room temperature, as shown in Figure 4d.

Thus, short carbon fiber has a strengthening effect on the tensile strength of the 2A12 aluminum alloy material and leads to a reduction in elongation after fracture.

### 3.2. Analysis of Strength Enhancement Mechanisms

Figure 3 presents the XRD patterns of 6 vol.% SCF-reinforced 2A12 composites tested at room temperature as well as at 180 °C and 220 °C. Compared with PDF#04-0787, the Aluminum (111) peak shifted from 38.58° to 38.42°, the (200) peak from 44.84° to 44.74°, the (220) peak from 65.2° to 65.14°, and the (311) peak from 78.32° to 78.28°. The composite had a preferential orientation in the (220) plane due to the hot extrusion. The signals of Al_4_C_3_ were not found in the XRD pattern. As the temperature rises, all the aluminum peaks shift to a lower degree. The peak shift indicates tensile residual stress in the matrix, which lowers the mechanical properties of the composites [26].

The strengthening effect of grain refinement for yield strength ($\Delta \sigma_{GR}$) can be calculated by the Hall–Petch relationship [27].

(2) $$\Delta \sigma_{GR}=k_{YS}(D_{c}^{-0.5}-D_{m}^{-0.5})$$

where $k_{YS}$ is the Hall–Petch slope for yield strength, about 0.04 MPa·m^1/2^ [28], and *D_c_* and *D_m_* are the grain sizes of Al for composite materials and unreinforced materials. The strengthening effect predicted by the Hall–Petch equation can be found in the change in Vickers hardness ($\Delta H_{GR}$), as

(3) $$\Delta H_{GR}=k_{H}(D_{c}^{-0.5}-D_{m}^{-0.5})$$

where $k_{H}$ is the Hall–Petch slope for hardness. The grain sizes can be calculated using Scherrer’s formula.

(4) $$D=\frac{K\lambda }{\beta cos\theta }$$

where *K* is the shape factor; *λ* is the X-ray wavelength, 0.15406 mm for Cu Kα; *β* is the full width at half maximum of the diffraction peak; and *θ* is the Bragg angle.

**Figure 5:**
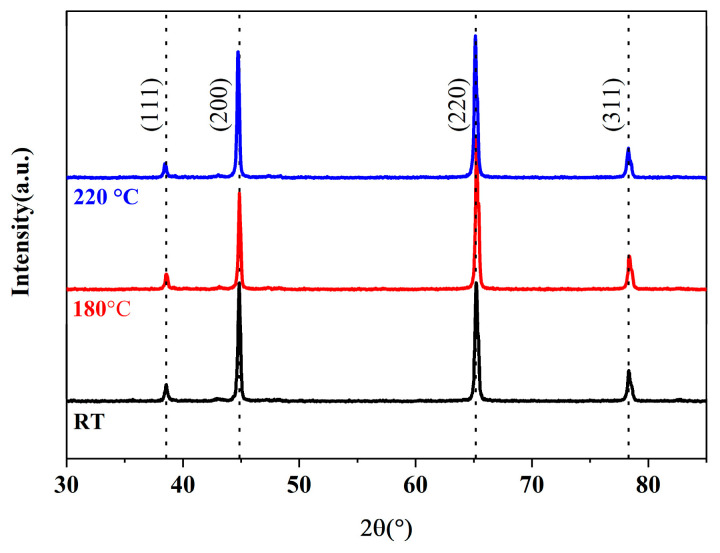
XRD pattern of 6 vol.% SCF-reinforced 2A12 composite. The black line represents the room temperature, the red line represents 180 °C, and the blue line represents 220 °C. The dash line is derived from PDF#04-0787.

Grain refinement has a positive effect on the composite, as all composites’ minimums exceed the hardness and yield strength of the 2A12. The results in Figure 5 show that grain sizes are close (about 28 nm), as reported in earlier findings [19], which means grain refinement has an impact on the mechanical properties of samples, but did not result in strength loss at high temperature.

**Figure 6:**
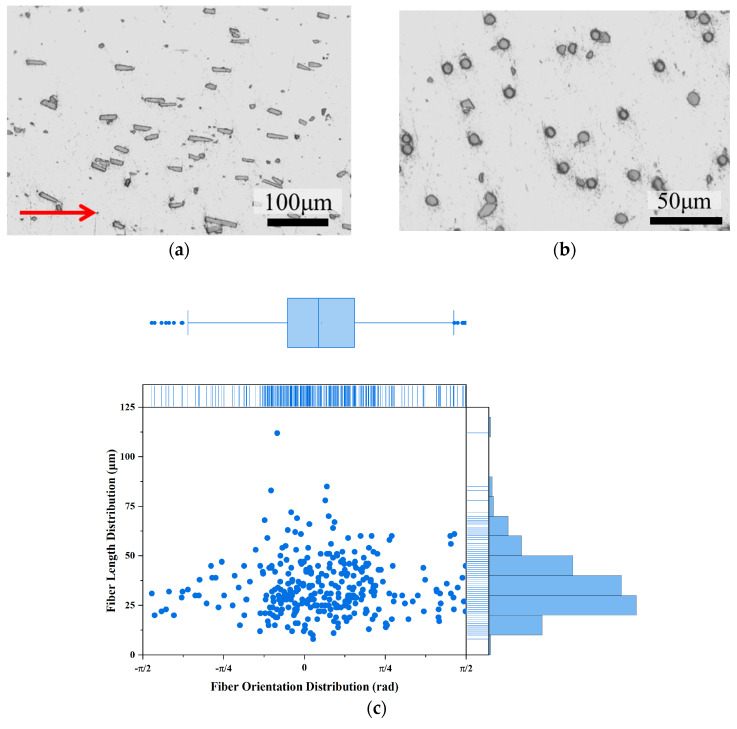
The optical surface morphology and fiber distribution of 6 vol.% SCF-reinforced 2A12 composite. (**a**) Longitudinal section; (**b**) transverse section; (**c**) fiber orientation and length distribution.

Figure 6 illustrates the common arrangement of short carbon fibers within the matrix of the as-extruded sample (6cf) along the longitudinal cross-section. The fibers are uniformly dispersed in the matrix and arranged in the direction of the extrusion flow (shown as the red arrow). The shapes of SCF predominantly exhibit a rectangular or oval form, as shown in Figure 6a. No obvious clustering of fiber was observed, and there were no voids in the matrix, as shown in Figure 6b. Their length distribution on the observed polished surface, with an average length of 33 µm, is depicted in Figure 6c. The shortening of the fibers is attributed to the sample preparation process, similar to the results reported in our previous studies [19]. The fiber angles generally align with the extrusion direction, and from the box range, more than 50% of the carbon fibers have a deviation angle range of π/4 (about 45°). In addition, carbon fibers with larger deviation angles have shorter lengths.

The theoretical strength of the composite by load transfer mechanism can be described as follows [29]:

(5) $$\sigma_{c}=\sigma_{f}V_{f}\frac{l}{l_{c}}+\sigma_{m}V_{m}\;,\;for\;l<l_{c}$$

(6) $$\sigma_{c}=\sigma_{f}V_{f}\left(1-\frac{l}{l_{c}}\right)+\sigma_{m}V_{m},\;\;for>l_{c}$$

where $\sigma_{f}$ and $\sigma_{m}$ are the strength of the fiber and matrix, and l is the fiber length. $l_{c}$ is the critical length of reinforcement [17], which is expressed as

(7) $$l_{c}=\frac{\sigma_{f}d}{2\tau_{M}}$$

where *τ_M_* is the shear strength of the matrix (considered as half of the tensile strength), *d* is the fiber diameter, which is 7 μm for carbon fiber. After calculation, $l_{c}$ lies in the range from about 169 µm to 249 µm, which is five times longer than the short carbon fiber in the extruded samples. Therefore, Formula (5) should be considered.

**Figure 7:**
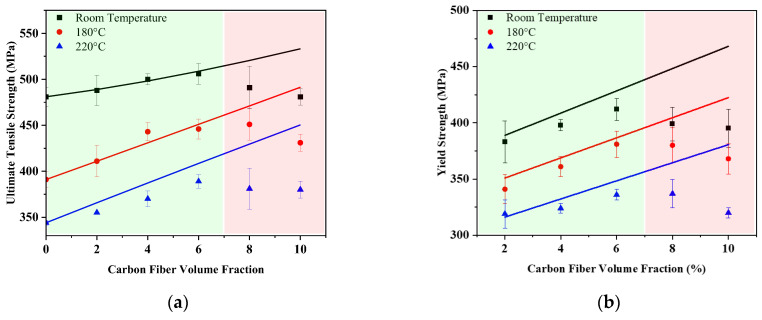
The theoretical strength and experimental strength of SCF/2A12 and 2A12 samples. (**a**) Ultimate tensile strength; (**b**) yielding strength. Solid lines are theoretical composite strength, and the black, red, and blue colors represent each temperature. The green background means small deviations, and the red background means large deviations.

The improvement from load transfer strengthening mechanisms also depends on the volume fraction of carbon fibers. Theoretical strengths of the composites at each temperature are calculated and shown as solid lines in Figure 7a,b. Comparing the theoretical values with the experimental results, one can find that the strength improvement is close to the theoretical value when the SCF content is less than 6% (with a green background). Further increasing SCF content (with a red background) results in a large deviation between the experimental value and theoretical calculation value.

**Figure 8:**
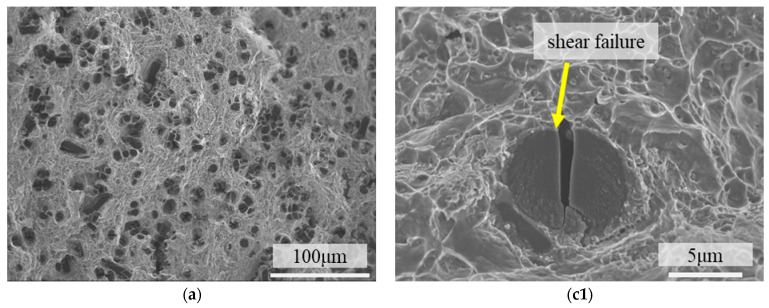

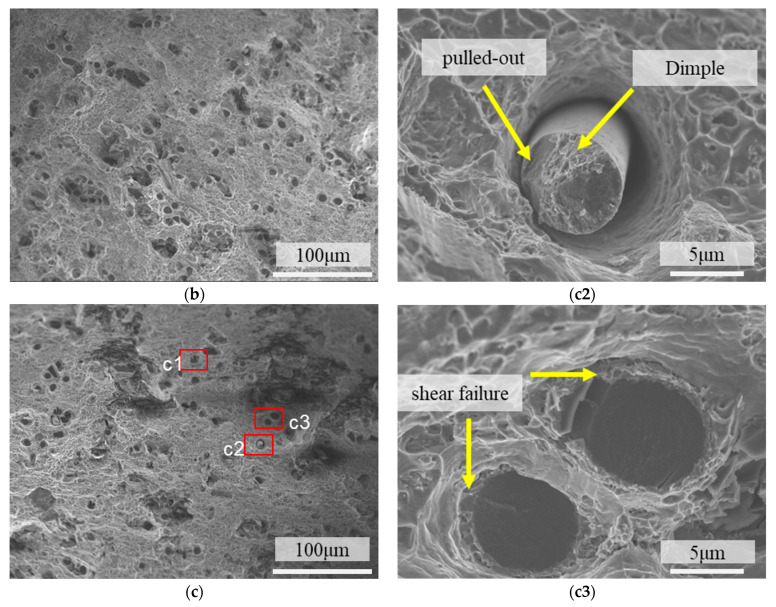
Fracture surface morphologies of 4 vol.% short-carbon-fiber-reinforced 2A12 composite. (**a**) Fractured at room temperature; (**b**) fractured at 180 °C; (**c**) fractured at 220 °C. (**c1**–**c3**) Magnified views of Figure 8c.

**Figure 9:**
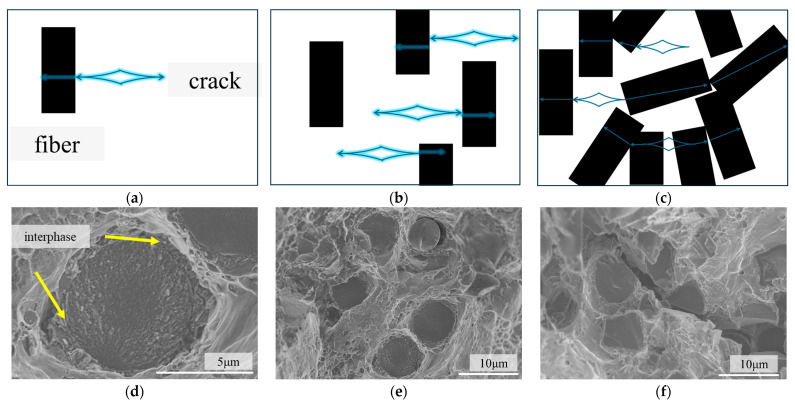
Schematic diagrams of the shear failure. (**a**) Fiber shear failure; (**b**) independent fiber shear failure; (**c**) continuous fiber shear failure; (**d**) related SEM graph of fiber shear failure; (**e**) related SEM graph of independent fiber shear failure; (**f**) related SEM graph of continuous fiber shear failure. The black cuboid represents carbon fiber. The blue diamond represents the crack. The blue arrow represents fracture surface.

Figure 8 shows the fracture morphologies of 4 vol.% short-carbon-fiber-reinforced AA2024 composites. Dimples can be seen in the matrix, and short carbon fibers are perpendicular to the fracture surface. The fibers have two contrast ratios in the image. The multiple failure modes were also observed in continuous carbon-fiber-reinforced 2A12 aluminum alloy [30]. Darker circles indicate fiber shear (see Figure 8(c1)), while lighter circles indicate that the fiber pulled out and underwent debonding (see Figure 8(c2)). There are some aluminum dimples clinging to the carbon fiber. Most carbon fiber fractures appear to be shear failure, suggesting strong interfacial bonding (see Figure 8a–c). The load transfer mechanism can be ensured by the interphase (such as Al_2_Cu, Al_4_C_3_ [31]), which is thrusting into the matrix, as shown in Figure 8(c1) and Figure 9d. The process of fiber failure serves to absorb external energy and facilitate the load transfer mechanism [32].

Compared with as-sintered samples in our earlier research [17], hot extrusion led to a more aligned carbon fiber distribution. In the as-sintered composites, the tensile strength peaks at 4 vol.%, while after extrusion, the tensile strength peaks at 6 vol.%.

### 3.3. Strength Reduction by Excess Carbon Fiber Addition

**Figure 10:**
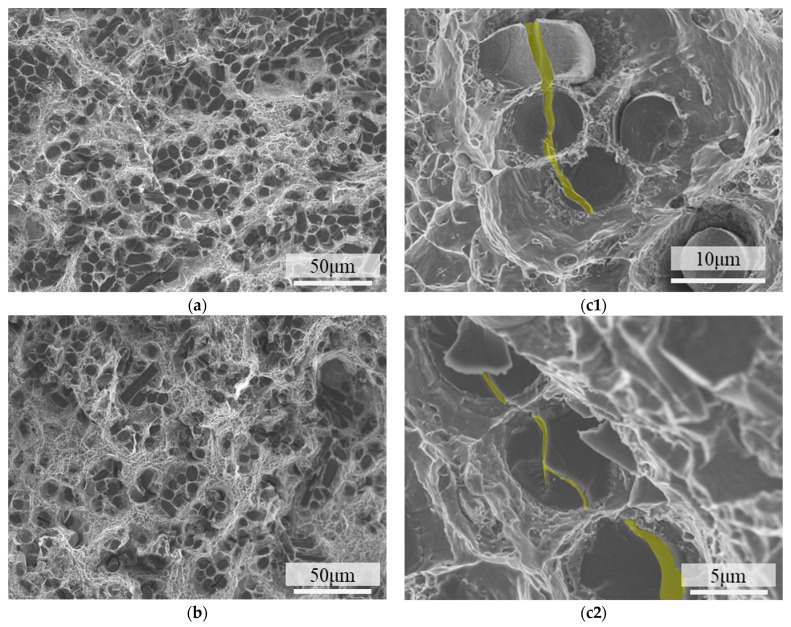

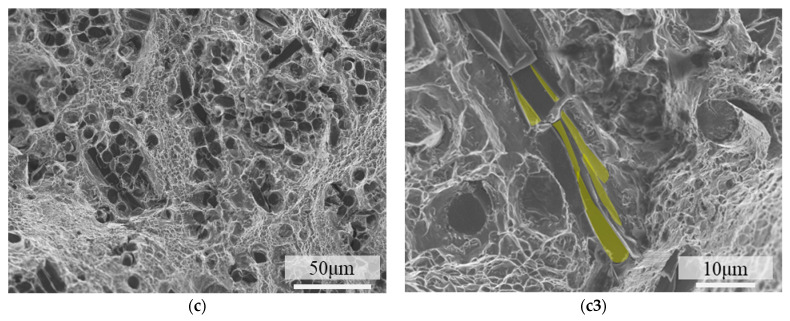
Fracture surface morphologies of 10 vol.% short-carbon-fiber-reinforced 2A12 composite. (**a**) Fractured at room temperature; (**b**) fractured at 180 °C; (**c**) fractured at 220 °C. (**c1**–**c3**) A magnified view of Figure 10c. The cracks that continuously shear the short carbon fibers are highlighted.

In the carbon fiber shear failure mode, the crack initiated in the matrix. Because of the strong interfacial bonding, the crack shears the carbon fiber without interface separation [20], as shown in Figure 9a,d. At low fiber volume fractions, carbon fiber shear occurs independently, as shown in Figure 9b,e. The cracks on the two adjacent carbon fibers are almost perpendicular to each other, as shown in Figure 8(c3). Compared with low-content samples, the distance between the SCF is significantly reduced when the volume fraction increases. Although the fibers are evenly dispersed in the matrix, contact between the fibers is unavoidable because the orientation of the fibers is not the same. Carbon fiber, as an anisotropic material, is not good at resisting the propagation of shear cracks. The failure of the adjacent carbon fibers may cause the external load to redistribute in the matrix [33], accelerating the crack propagation. Therefore, when the space between the SCF is too small, adjacent fibers can easily be cut off by the pre-existing crack [34]. Continuous fiber shear failure can be seen in the fracture surface of composites with 10cf, as shown in Figure 10. This phenomenon is temperature-independent, as shown in Figure 10a–c, for samples examined at three temperatures. No matter what the relative angle of the fibers is, fibers are likely to share the same crack. Randomly select and magnify the samples at 220 °C for observation. Figure 10(c1,c2) show three carbon fibers normal to the fiber cross-section, while Figure 10(c3) demonstrates fibers normal or parallel to the tensile cross-section.

The proportion of continuously broken fibers ($P_{cb}$) is calculated as

(8) $$P_{cb}=\frac{N_{cb}}{N}$$

where $N_{cb}$ is the number of continuously broken fibers, and N is the number of all fibers in the same SEM figure.

**Figure 11:**
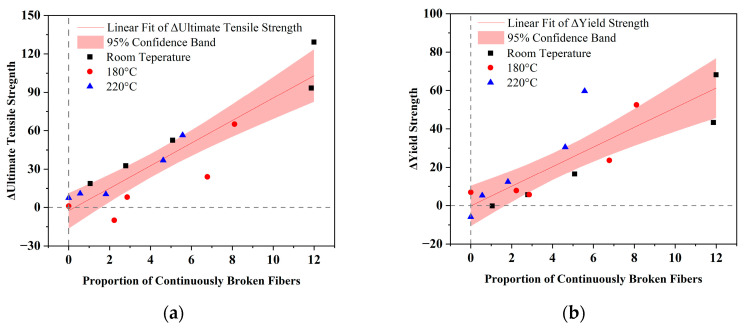
The relationship between the proportion of continuously broken fibers and reinforcement effects at different temperatures. (**a**) Deviation of ultimate tensile strength; (**b**) deviation of yield strength. The red line represents the linear fit of ΔUltimate Tensile Strength or ΔYield strength, and the red band indicates the 95% confidence interval of the linear fit.

Figure 11 illustrates how the percentage of continuously fractured fibers correlates with the deviation from the theoretical strength. The black square, red circle, and blue triangle represent the strength differences of composites at room temperature, 180 °C, and 220 °C, respectively. The linear fit values of ΔUltimate Tensile Strength and ΔYield strength are calculated regardless of temperature. Both linear fit lines have a small interception, as shown in Figure 11a,b, and most points are close to 95% confidence bands. A strong positive linear relationship between fiber and ultimate tensile strength is revealed in Figure 11a, as indicated by a Pearson correlation coefficient of 0.91 and an R-square value of 0.83. The linear relationship is weaker for yield strength, with a Pearson correlation coefficient of 0.86 and an R-square value of 0.74.

## 4. Conclusions

The 2A12 aluminum alloy reinforced with aligned short carbon fibers was fabricated via powder metallurgy. The incorporation of short carbon fibers enhanced both the hardness and tensile strength of the matrix while simultaneously decreasing the overall material weight.

(1) The composite achieves its optimal performance in hardness, ultimate tensile strength, and yield strength with the addition of 6 vol.% short carbon fibers. The yield strength increases by 16.4% from 354 MPa to 412 MPa at room temperature, by 18.7% from 321 MPa to 381 MPa at 180 °C, and by 15.8% from 290 MPa to 336 MPa.
(2) Grain refinement can characterize the trend in mechanical properties of the composite at room temperature, while the load transfer mechanism can describe its behavior across different testing temperatures. The performance of composite materials decreases after the volume fraction of carbon fibers exceeds 6%, due to continuous breaking. The discrepancy observed between theoretical predictions and measured composite strengths appears to be influenced by the extent of continuous fiber fragmentation.
(3) Adding short carbon fiber successfully extends the service condition of the 2A12 aluminum alloy by approximately 40°C. The yield strength of the 6 vol.% short-carbon-fiber-reinforced 2A12 aluminum alloy at 220 °C is comparable to that of the unreinforced 2A12 aluminum alloy at 180 °C, even higher than the minimum value of the material at 20 °C in the ISO standard.

The performance of the material and the preparation method both meet the application requirements of aluminum alloy drill pipes, possess certain market value, and can also be used in other high-temperature lightweight working conditions.

## Figures and Tables

**Table 1:** Chemical composition of 2A12 alloy (wt. %).

Element	Cu	Mg	Mn	Fe	Si	Zn	Cr	Ti	Al
Content	4.59	1.68	0.43	0.060	0.043	<0.05	<0.01	<0.001	Bal.

**Table 2:** The density and hardness of 2A12 and composites.

Abbreviation	Volume Fraction (%)	Measured Density (g/cm^3^)	Relative Density (%)	Hardness (HV)
2A12	0	2.756 ± 0.006	99.4	122.5 ± 9.7
2cf	2	2.742 ± 0.003	99.5	125.3 ± 3.8
4cf	4	2.722 ± 0.004	99.5	136.0 ± 6.9
6cf	6	2.704 ± 0.002	99.6	155.4 ± 16.4
8cf	8	2.683 ± 0.008	99.5	141.8 ± 10.7
10cf	10	2.665 ± 0.003	99.6	145.6 ± 9.6

## Data Availability

The original contributions presented in this study are included in the article. Further inquiries can be directed to the corresponding authors.
